# Recent advances in synthetic biosafety

**DOI:** 10.12688/f1000research.8365.1

**Published:** 2016-08-31

**Authors:** Anna J. Simon, Andrew D. Ellington

**Affiliations:** 1 Department of Chemistry & Biochemistry, University of Texas at Austin, Austin, TX, 78712, USA

**Keywords:** synthetic organism, engineered microbe, applied microbiology

## Abstract

Synthetically engineered organisms hold promise for a broad range of medical, environmental, and industrial applications. Organisms can potentially be designed, for example, for the inexpensive and environmentally benign synthesis of pharmaceuticals and industrial chemicals, for the cleanup of environmental pollutants, and potentially even for biomedical applications such as the targeting of specific diseases or tissues. However, the use of synthetically engineered organisms comes with several reasonable safety concerns, one of which is that the organisms or their genes could escape their intended habitats and cause environmental disruption. Here we review key recent developments in this emerging field of synthetic biocontainment and discuss further developments that might be necessary for the widespread use of synthetic organisms. Specifically, we discuss the history and modern development of three strategies for the containment of synthetic microbes: addiction to an exogenously supplied ligand; self-killing outside of a designated environment; and self-destroying encoded DNA circuitry outside of a designated environment.

## Strategy 1: addiction

**Figure 1.**
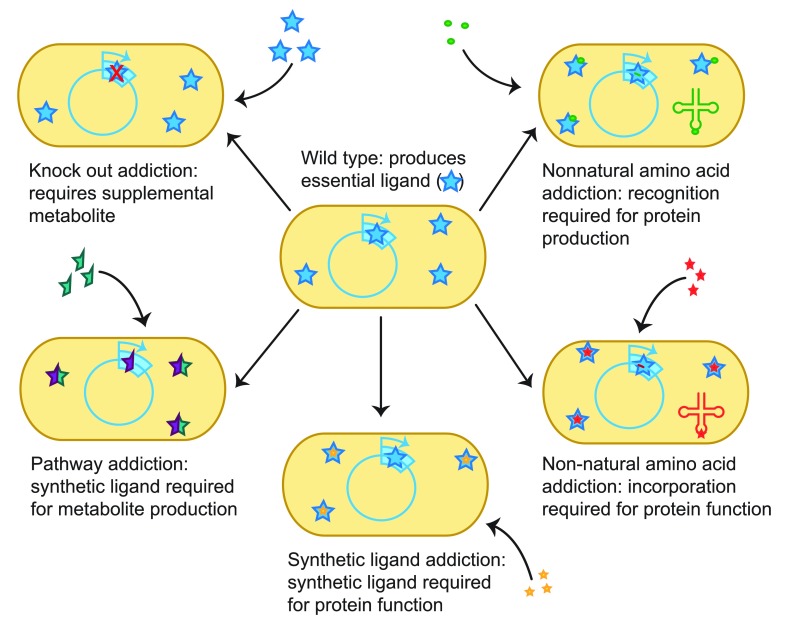
A common strategy for the containment of synthetic organisms is to engineer them to require an exogenously supplied ligand. Common methods for achieving this include (clockwise from top left) knocking out a required gene and exogenously supplying the gene product ^1^, requiring the amber-mediated incorporation of a synthetic amino acid for essential protein production ^9^, requiring the amber-mediated incorporation of a synthetic amino acid for essential protein function ^2, 7^, requiring a synthetic molecule as a cofactor for protein function ^23^, requiring a synthetic nucleotide for essential gene replication or translation, and requiring a synthetic molecule as a precursor for a key metabolite ^24^.

As with the lysine-deficient fictional dinosaurs in the book *Jurassic Park*, it may be possible to employ addiction strategies to make it difficult for organisms to survive outside of their designated habitats. This biocontainment strategy dates back to the earliest days of cloning with the development of the specialized *Escherichia coli* strain χ1776, which lacked functional aspartate-semialdehyde dehydrogenase ( *asd*), (L-delta-1-tetrahydrodipicolinate synthetase) ( *dapD*) *,* and thymidylate synthetase ( *thyA*) genes and thus required their products, diaminopimelic acid (DAP) and thymine or thymidine, to survive ^1^. Because this strategy is so straightforward—requiring only the creation of organisms deficient in the ability to produce a key metabolite and this is readily achieved by targeted and random mutagenesis—it has remained commonly employed and integrated into more sophisticated synthetic biosafety platforms through recent times ^2, 3^ ( Figure 1, top left).

Despite the straightforwardness of this simple addiction strategy, it was not sufficient to contain *Jurassic Park*’s resurrected fictional dinosaurs because of the ready availability of lysine in the environment, and real-life organisms addicted to natural chemicals could likely escape the same way. Wright and colleagues, for example, demonstrated that Δ *thyA* mutants grew readily in media lacking specifically supplied thymidine but supplemented with a small amount of sterilized soil, demonstrating that environmental nutrients may complement metabolic deficiencies ^3^. Furthermore, the engineered inability to generate a key metabolite often inhibits the growth of an organism even when the metabolite is heavily supplemented, rendering such engineered organisms ill-suited for use in bioreactors and many other applications ^1, 4^. Thus, the more modern version of this strategy is to engineer organisms that depend not on a natural compound but on a synthetic one. If engineered organisms require a synthetic compound for survival, they will be unable to survive outside of a laboratory or other highly specialized environment in which the chemical is supplied. However, this engineering feat is more difficult than simply generating organisms with a “broken” ability to produce a naturally occurring metabolite. Instead, it requires engineering some sort of metabolic or functional connection to a synthetic chemical that was previously irrelevant to the organism’s biology.

One strategy by which researchers have adapted organismal metabolism to require synthetic compounds is by altering the genetic code to incorporate and even require unnatural amino acids. In this regard, the development of so-called orthogonal translation machinery has advanced to the point where the incorporation of unnatural amino acids into proteins, at least across from amber codons, is relatively straightforward ^5^. Although an organism can readily be rendered dependent upon suppression of an amber codon (for example, by introducing the amber codon into an essential protein), rendering a protein (and thus its organism) dependent on a specific synthetic amino acid is considerably more complicated, as it requires the specific, introduced amino acid to be necessary for protein function ( Figure 1, top right).

Addicting a protein to a synthetic amino acid can potentially be achieved by either design or selection. On the design side, Church and colleagues engineered the essential adenylate kinase and tyrosyl-tRNA synthetase proteins to require the synthetic L-4′4′-biphenylalanine amino acid in their hydrophobic cores to stably fold and thus function ^2^. Combining this synthetic amino acid requirement with the classic DAP requirement employed in the χ1776 strain ^1^ produced organisms that escape their designated environment (that is, grow in the absence of their exogenous ligands) with frequencies of less than 6.4 × 10 ^–11^. Notably, this level of containment is considerably tighter than the National Institutes of Health (NIH)-suggested maximum escape frequency of 10 ^−8^ ^6^. On the selection side, Ellington and colleagues evolved the essential β-lactam antibiotic resistance protein TEM β-lactamase to require either of the synthetic amino acids 3-nitro-L-tyrosine (3nY) or 3-iodo-L-tyrosine (3iY), achieving escape frequencies of as low as less than 5 × 10 ^−11^ ^7^. They further demonstrated the generality of this synthetic amino acid-addicted protein by demonstrating that the evolved 3nY addicted protein retained its addiction in the diverse bacterial species *E. coli*, *Shigella flexneri*, *Salmonella enterica*, *Yersinia ruckeri*, and *Acinetobacter baylyi*.

Orthogonal translation machinery and synthetic amino acid incorporation become especially useful in the context of organisms with skewed genetic codes, such as the recoded GRO *E. coli* strain developed by Isaacs and colleagues ^8^. This strain was exhaustively engineered to completely lack the amber (TAG) stop codon and may now be readily engineered to rely on unnatural amino acids and their cognate tRNAs that recognize ambers inserted into essential genes ( Figure 1, center right). In addition to their use of a GRO strain to express rationally designed essential proteins requiring a synthetic amino acid, as discussed above ^2^, Rovner and colleagues explored this strategy in a more high-throughput way by employing multiplex automated genome engineering (MAGE) to introduce amber mutations into conserved and functional aromatic residues in 22 essential proteins in a GRO strain containing a *Methanocaldococcus jannaschii* tRNA:aminoacyl-tRNA synthetase pair, which in turn translated the amber codons to synthetic phenylalanine-derived amino acids ^9^. Several strains containing a single variant protein exhibited near-normal growth in media containing the synthetic amino acids but escape frequencies of less than about 10 ^−6^ in non-supplemented media. Combining several mutations further reduced this escape rate; notably, a strain containing mutations in essential residues of the MurG, DnaA, and SerS proteins grew normally in supplemented media but “escaped” with rates below the detectable 4 × 10 ^−11^ frequency after 20 days of growth ^9^. This very low escape frequency arises from the dependence of multiple proteins on these synthetic amino acids rather than the dependence *per se* of a single protein: whereas the probability of one amber codon reverting to a natural codon is moderate (~10 ^−7^), the probability of getting three in parallel is much lower.

A strategy to render organisms dependent on synthetic molecules at an even deeper level than addicting them to synthetic tRNAs or amino acids is to addict them to synthetic nucleotides. Mutzel and colleagues demonstrated the ability to engineer organisms that heavily incorporate such synthetic nucleotides into their genomes by evolving *E. coli* strains that can substitute the synthetic nucleotide analog 5-chlorodeoxyuridine for deoxythymidine in their genomes ^10^. To do this, they first engineered a thymidine synthase-deficient strain of *E. coli* containing the *Lactobacillus leichmannii* nucleoside deoxyribosyltransferase gene, enabling it to survive in low concentrations of thymine and convert exogenous 5-chlorouracil to its nucleotide analog. Next, the authors selected for mutants capable of substituting 5-chlorodeoxyuridine for genomic thymine by stressing the cells with media containing an increasingly high proportion of 5-chlorouracil relative to thymine. This produced organisms containing mutations enabling them to substitute 5-chlorodeoxyuridine in place of 90% of their genomic deoxythymidines and to grow equally well on 5-chlorouracil- and thymine-containing solid. Although these organisms were not addicted to 5-chlorodeoxyuridine *per se*, as they retained their native ability to use thymine, this work demonstrated the ability of organisms to efficiently substitute synthetic base pairs in their genome. Conceivably, this work could be expanded to develop organisms with the ability not just to substitute a natural nucleotide for a synthetic analog but to require it.

Although it is possible to imagine the development of an organism addicted to a single synthetic nucleotide, a more airtight strategy would likely be to engineer organisms addicted not just to a single synthetic nucleotide but to a complementary pair ( Figure 1, bottom left). This synthetic base pair could likely be further employed to encode synthetic amino acids (such as the examples described above) required for the function of an essential protein, thus addicting the organism at multiple genetic levels. To date, multiple groups have developed such synthetic base pairs and demonstrated their robust function and replication *in vitro*. For example, Hirao and colleagues developed another synthetic base pair, 7-(2-thienyl)-imidazo[4,5-b]pyridine (“Ds”) and 2-nitro-4-propynylpyrrole (“Px”) ^11^, effectively amplified by polymerase chain reaction (PCR) amplification ^12, 13^. Benner and colleagues developed both the deoxyribo and ribonucleoside forms of a base-pairing set of synthetic nucleotides, 6-amino-5-nitro-3-(1′-β-d-2′-deoxyribofuranosyl)-2(1H)-pyridone (“Z”) and 2-amino-8-(1′-β-d-2′-deoxyribofuranosyl)-imidazo[1,2-a]-1,3,5-triazin-4(8H)-one (“P”), that are effectively incorporated into a double helix ^14^, replicated by PCR ^15^, and transcribed by T7 RNA polymerase ^16^. Romesberg and colleagues developed a separate set of synthetic base pairs, d5SICS-dMMO2 ^17^ and d5SICS-dNAM ^18^, that likewise are effectively amplified in PCR ^19^ and T7 RNA polymerase *in vitro* ^20^ and, in a highly notable advance, specifically and relatively stably incorporated and maintained into plasmids replicated in *E. coli* over multiple generations and faithful replication *in vivo* in *E. coli* for several generations (>15 hours of growth) ^21^.

Unnatural nucleotides and amino acids are not the only possible compounds to use for addicting biochemistry. Dependence on unnatural vitamins, cofactors, or other metabolites could also prevent growth outside of a specialized environment. In an early example of the small-molecule addiction strategy, Schultz and colleagues engineered a mutant interface between human growth hormone and its receptor containing an interfacial cavity that required binding by the synthetic cofactor 5-chloro-2-trichloromethylimidazole for functional ligand-receptor interactions ^22^. Though not explicitly intended for synthetic biosafety applications, cells containing this mutant pair exhibited a more than 1,000-fold greater response in the presence of the synthetic molecule, suggesting that this approach of engineering synthetic molecule-dependent ligand–receptor pairs could be a useful means by which to restrict their function to a specific environment ^22^. More recently, researchers have integrated this cofactor addiction strategy with computational and selection methods to readily produce proteins dependent on synthetic molecules for function. For example, Lopez and Anderson engineered benzothiazole-dependent “SLiDE” mutant proteins for the essential genes for phenylalanine tRNA synthetase, tyrosyl tRNA synthetase, methionyl tRNA synthetase, DNA polymerase III, and adenylate kinase, which require the synthetic ligand benzothiazole to bind as a cofactor to stabilize the hydrophobic core and thus the folded, functional form of the protein ^23^ ( Figure 1, bottom right). Strains containing three such mutants in parallel achieved escape frequencies of less than 3 × 10 ^−11^ after two days in culture.

An alternative to addicting biomolecules to a synthetic chemical is to instead addict a biochemical pathway. This is achieved by modifying such pathways to convert an exogenously supplied synthetic compound into a required, otherwise-unavailable metabolite, thus addicting the organism to that molecule. For example, Quandt and colleagues engineered *E. coli* to require caffeine by first knocking out an essential enzyme in the guanine synthesis pathway, inosine-5′-phosphate dehydrogenase, that catalyzes the formation of the key intermediate xanthosine-5′-phosphate from inosine-5′-phosphate ^24^. They then introduced a refactored *Pseudomonas putida* alkylxanthine degradation pathway and a *Janthinobacterium marseille* glutathione S-transferase to demethylate caffeine into xanthine, which the strain then converts to xanthosine-5′-phosphate and in turn guanine ( Figure 1, center left). Although they did not explicitly apply this study to synthetic biosafety, the authors demonstrated that cell growth was severely limited in the absence of caffeine, suggesting an ability to contain these organisms to a caffeine-rich environment. Although the strategy of addicting organisms to an exogenous, synthetic substrate via pathway refactoring has seen little exploration as a synthetic biosafety strategy, technologies for refactoring metabolic pathways are fairly well developed ^25, 26^, suggesting that this approach could prove rather straightforward.

## Strategy 2: kill switches

In addition to engineering synthetic organisms that depend passively on specific, synthetic environmental molecules to function, researchers have engineered synthetic organisms that actively kill themselves outside of their designated environments. Nature employs a similar strategy: many bacteria contain specific toxin:anti-toxin pairs that lead to selfish episome retention ^27^. If the anti-toxin activity is lost (that is, through loss of a plasmid carrying the anti-toxin), the toxin will kill the cell.

**Figure 2.**
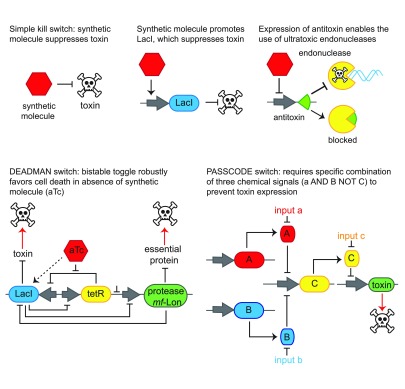
“Kill” switches activate cell-killing proteins in the absence of specific molecular cues. Simple, early kill-switch architectures commonly used include a switch in which a synthetic molecule directly represses the expression of a toxin (top left) ^28^ and a switch in which a synthetic molecule drives LacI expression, which in turn represses toxin expression (top center) ^29^. The expression of anti-toxins along with their cognate endonuclease anti-toxin has enabled the use of endonucleases in such kill switches, which destroy DNA in addition to simply killing cells (top right) ^34, 35^. Recently, Chan and colleagues developed ultra-robust kill switches ^31^. Their DEADMAN switch (bottom left) is a bistable switch that robustly activates two cell-killing modalities in the absence of a synthetic signal molecule, and their PASSCODE switch (bottom right) requires a specific combination of three synthetic molecules to block the production of a cell-killing toxin.

The first synthetic, self-killing organisms employed these toxins in simple kill switches, in which exogenously supplied small molecules repressed toxin expression. In the absence of these effectors, the cell would express the toxin, killing itself. In one of the first such studies, Andersson and colleagues expressed the membrane-depolarizing toxin hok gene under the tryptophan-repressible trp promoter in *E. coli* ^28^. In the absence of a high concentration of tryptophan supplied in media, the organisms would express hok, thus killing themselves ( Figure 2, top left). Later, several authors employed LacI-based inverters to construct more modular kill switches activated by the absence or presence of a broader range of synthetic molecules. For example, Ramos and colleagues developed a kill switch in *P. putida* in which the 3-methylbenzoate-activated TOL promoter drove production of LacI, which in turn repressed the toxin gef ^29^ ( Figure 2, top center). An absence of 3-methylbenzoate turned off LacI expression and thus gef repression, resulting in gef expression-mediated cell death. Genes that sequester key metabolites, starving the cells, may be employed as an alternative to toxins. For example, Cantor and colleagues ^30^ demonstrated a switch similar to that of Andersson and colleagues in which the killing modality is the overexpression of streptavidin, which binds and sequesters the key metabolite biotin, leading to cell death. Although these single-component kill switches are generally robust, they are subject to failure due to point mutations inactivating the killing mechanism, which generally occur at frequencies of 10 ^−3^ to 10 ^−7^ ^4^.

As with the improvements to addiction-based synthetic biosafety strategies in which multiple dependencies are grouped together to ensure low reversion, recent advancements have enabled the development of multilayered kill switches that are both more robust and more dependent on specialized (artificial) environments. Notably, Collins and colleagues developed several architectures for highly robust kill switches consisting of networks of multiple component switches that interact to reinforce the “killing” state in the absence of a strong, highly specific “don’t kill” environmental signal, providing backup in case one component is mutated or otherwise non-functional ^31^. Specifically, their “DEADMAN” switch employs a bistable regulator with mutually reinforcing feedback loops to actively drive both the expression of a toxin and the degradation of an essential cell protein in the absence of a specific effector ( Figure 2, bottom left). Likewise, their “PASSCODE” switch requires the presence and absence of a specific combination of synthetic effectors (that is, an AND/NOT gate) to repress toxin expression ( Figure 2, bottom right). In the absence of these specific inputs, these networked switches achieved escape frequencies below the detectable limit of 10 ^−7^ ^31^. Although the reported escape frequencies do not explicitly improve upon those of previously reported kill switches (that is, 30) or addiction strategies ^7, 8, 23^, these architectures presumably would show improved stability due to their bistability, resulting in quicker, more complete killing.

## Strategy 3: self-destroying

Although the escape of live organisms represents the most obvious hazard in synthetic biology, the possibility also exists that synthetic DNA could be released from even a dead cell and make its way into the environment via natural gene transfer ^3, 32^. Thus, to make truly well-contained synthetic organisms or ecologies, it is important not just to kill escaping organisms but additionally to destroy their DNA.

A convenient strategy to simultaneously kill cells and destroy their genes is to employ nucleases as toxins in cellular kill switches such as those described above. Early studies demonstrated the power of these nuclease-based kill switches. For example, in a 1994 proof of concept, Ahrenholtz and colleagues demonstrated a heat-responsive nuclease-driven kill switch by driving the production of the *nuc* nuclease gene from *Serratia marcescens* with a thermoresponsive pL promoter in *E. coli* ^33^. Inducing by heating to 42°C killed cells with an escape rate after 2.5 hours of heating of 2 × 10 ^−5^. However, although this idea of employing nucleases as kill-switch toxins was explored in the early days of synthetic biosafety, their high toxicity required very low expression levels in the “off” state, which is difficult with chemically driven promoters ^4^. Consequently, nucleases have seen most biosafety use to prevent the dissemination of plasmids.

Torres and colleagues, for example, employed nuclease-mediated toxin:anti-toxin strategies to prevent plasmids from spreading through wild populations ( Figure 2, top right). Specifically, they co-expressed the nuclease EcoRI, encoded on a plasmid, and its cognate inhibitor, EcoRI methylase, encoded genomically ^34^. Because the inhibitor could be expressed at high levels, it counteracted the potentially leaky expression of the toxin. Should the plasmid be transferred, EcoRI expression should quickly destroy its circular form as well as dicing the new host chromosome (although small linear fragments might still be transferred between cells). Later authors adapted these nuclease–inhibitor pairs to function in circuits similar to those described in strategy 2: Gallagher, Isaacs, and colleagues developed a system which expressed EcoRI constitutively and the inhibitor methylase under an aTc-responsive promoter ^35^. In the absence of aTc, this single-layer switch achieved an escape frequency of approximately equal to 2.4 × 10 ^−6^. Most recently, the “DEADMAN” and “PASSCODE” switches employed EcoRI as a cell-killing toxin without coexpression of the inhibitor, as their bistability multiple reinforcing layers enabled the tight control of EcoRI expression ^31^.

Developments in genome editing, specifically by Cas9 and related systems, provide an alternative strategy for the direct removal and destruction of genes in response to environmental cues. For example, Caliando and Voigt describe a system termed “DNAi”, in which a genetically encoded, Cas9-containing circuit degrades specific sections of DNA in response to a molecular effector ^36^. Presumably, this circuit could be generalized to respond to the absence of a synthetic effector, leading to destruction outside of a laboratory environment. The DNAi system may be employed to target either plasmids (while not necessarily killing the cells) or the cell’s genomic DNA, including regions necessary for viability. The authors achieved degradation of both plasmid and genomic target regions with escape frequencies of less than 10 ^−8^.

## Future directions

The development of synthetic biosafety techniques that employ addiction, kill switches, and self-destroying modalities has now provided a framework for the development of “safe” synthetic organisms and ecosystems. Recent advances in synthetic biology, particularly in the manipulation of organisms’ genomes, the development of artificial biomolecules via rational and evolutionary design, and the construction of robust genetic switches, have in particular enabled the construction of robust safety features with measured escape frequencies well below those suggested by the NIH.

However, although the construction of effective biosafety mechanisms is underway, the broader question remains: how well will they work in real-world settings? The escape of even a single, errant bacterium could have widespread consequences, so it is crucial that these safety mechanisms be well explored. Justifiably, recent attention has turned from the construction of switches to understanding their potential failure modes, particularly in the context of the “real” world of complex physical environments and microbial communities outside of the laboratory. To this end, researchers have begun the systematic study of how the function and robustness of synthetic biological circuits, including the kill switches discussed here, depend on their environmental context. In an early study, Moser, Voight, and colleagues measured the performance (via GFP output) of an AND gate and a NOR gate under different media chemistries, bacterial strains, and reaction scales ^37^. They found that whereas the NOR gate’s function output was essentially independent of these changing conditions, the output of the AND gate varied nearly 20-fold over the range of growth conditions. A similar study that measured the dependence of escape rates of organisms containing the above-described biosafety “devices” on environmental conditions would shed needed light on the environmental robustness of these features.

As effective as genetically encoded safeguards might be, there are always unexpected consequences and therefore it is important to ensure that “dinosaur escape”, “black swan”, or “Grey goo” cataclysms, however unlikely, cannot occur. One way to study these potential failure modes would be to simulate them on a microbe-containing lab-on-a-chip environment such as the microflora-containing “gut on a chip” developed by Ingber and colleagues ^38^.These model environments could be employed to measure the escape frequency of synthetic organisms or DNA into more complex physical and biological environments, such as a community of other microbes, and what the evolutionary stability of engineered organisms and communities is over time.

Finally, there is now the possibility that an escaped, engineered organism can be hunted down … by other engineered organisms. That is, recently developed genome editing techniques have yielded so-called “gene drives”, genetic constructs that home to and overwrite themselves at homologous loci. Church and colleagues demonstrated the ability of synthetic gene drives to overwrite genes in both laboratory strain and wild-type yeast populations, specifically converting the wild-type *ADE2* gene (encoding phosphoribosylaminoimidazole carboxylase) to a mutant *ade2* variant. They mated haploid yeast containing the *ade2* gene drive with wild-type ( *ADE2*) haploid yeast of the opposite mating type and found that, while all of the resulting diploids initially inherited copies of both *ADE2* and *ade2*, virtually all (>99%) of their successive haploid progeny carried *ade2* (rather than 50% carrying each variant as would be expected), demonstrating successful overwriting of the wild-type *ADE2* genotype. The authors further demonstrated the capability of such gene drives to overwrite a second, essential gene ( *ABD1*) and to bias the inheritance of a cargo gene carried in cis with the gene overwritten by the gene drive ^39^. Although this example relies on sexual mating, in an asexual population, gene drives may likewise spread through horizontally transmitted genetic elements, such as broad host range vectors or phage to target specific genes or even the organisms containing them. Citorek and colleagues, for example, developed a phage-transmitted CRISPR/Cas9-based guided nuclease platform to target and cleave specific gene sequences, either destroying the plasmid on which they were located or introducing cytotoxic genomic double-strand breaks. They demonstrated successful targeting of bacteria containing the broad-spectrum antibiotic resistance gene New Delhi metallo-β-lactamase 1, reducing the viability of bacteria containing it by nearly 1,000-fold ^40^. This approach could presumably be applied to erase synthetic genes or organisms that had escaped their designated environments into the surrounding communities. Although gene drives and phage-transmitted gene destruction machinery are viewed as potentially harmful in their own right, the notion of self-propagating “code” that can repair other “code” is prevalent in the software community and eventually may prove tractable for biotechnology as well. For example, a transient gene drive “sweep” through a fermentor might prevent the unprogrammed escape of genetic material into non-engineered organisms.
